# A Review of Existing and New Treatments for the Management of Hand Eczema

**DOI:** 10.1177/12034754231188325

**Published:** 2023-07-27

**Authors:** Jessica S. S. Ho, Sonja Molin

**Affiliations:** 1 4257 Queen’s University, School of Medicine, Kingston, ON, Canada; 2 4257 Division of Dermatology, Department of Medicine, Queen’s University, Kingston, ON, Canada

**Keywords:** dermatology, hand eczema, therapeutics, hand dermatitis

## Abstract

Hand eczema is a chronic condition that affects an estimated 14.5% of the general population. It has severe quality of life ramifications in those that struggle with it, including days missed from work or school, productivity loss and impaired work functioning. For years, the standard of care included topical moisturizing creams, topical steroids and more recently systemic agents. As new therapeutic targets emerge and recent advances are being developed, it is now more possible than ever that hand eczema can be managed via the underlying mechanisms. A review of the literature was conducted to identify current treatment options for hand eczema and chronic hand eczema. The terms ‘hand eczema’, ‘hand dermatitis’ were used to search PubMed, CENTRAL and Embase. To identify new therapies still undergoing investigation, we used the terms ‘hand eczema’, ‘hand dermatitis’, ‘atopic dermatitis’, and ‘vesicular eczema of hands and/or feet’ to search Clinicaltrials.gov for all studies until December 2022. There were 56 ongoing clinical trials identified for pharmacological treatments for hand eczema on Clinicaltrials.gov from 2000 - 2022, with 16 that are new or ongoing. These included studies for dupilumab, ruxolitinib, delgocitinib (LEO124249), gusacitinib (ASN002), AFX 5931, and roflumilast (ARQ-252). Two major classes of drugs emerging for the treatment of hand eczema include IL-4/IL-13 inhibitors and JAK inhibitors. With the increase in efficacy seen with these new drugs, we are also noting improved adverse effect profiles, making them attractive options to add to a clinician’s management toolbox for patients with hand eczema.

## Introduction

**Figure 1:**
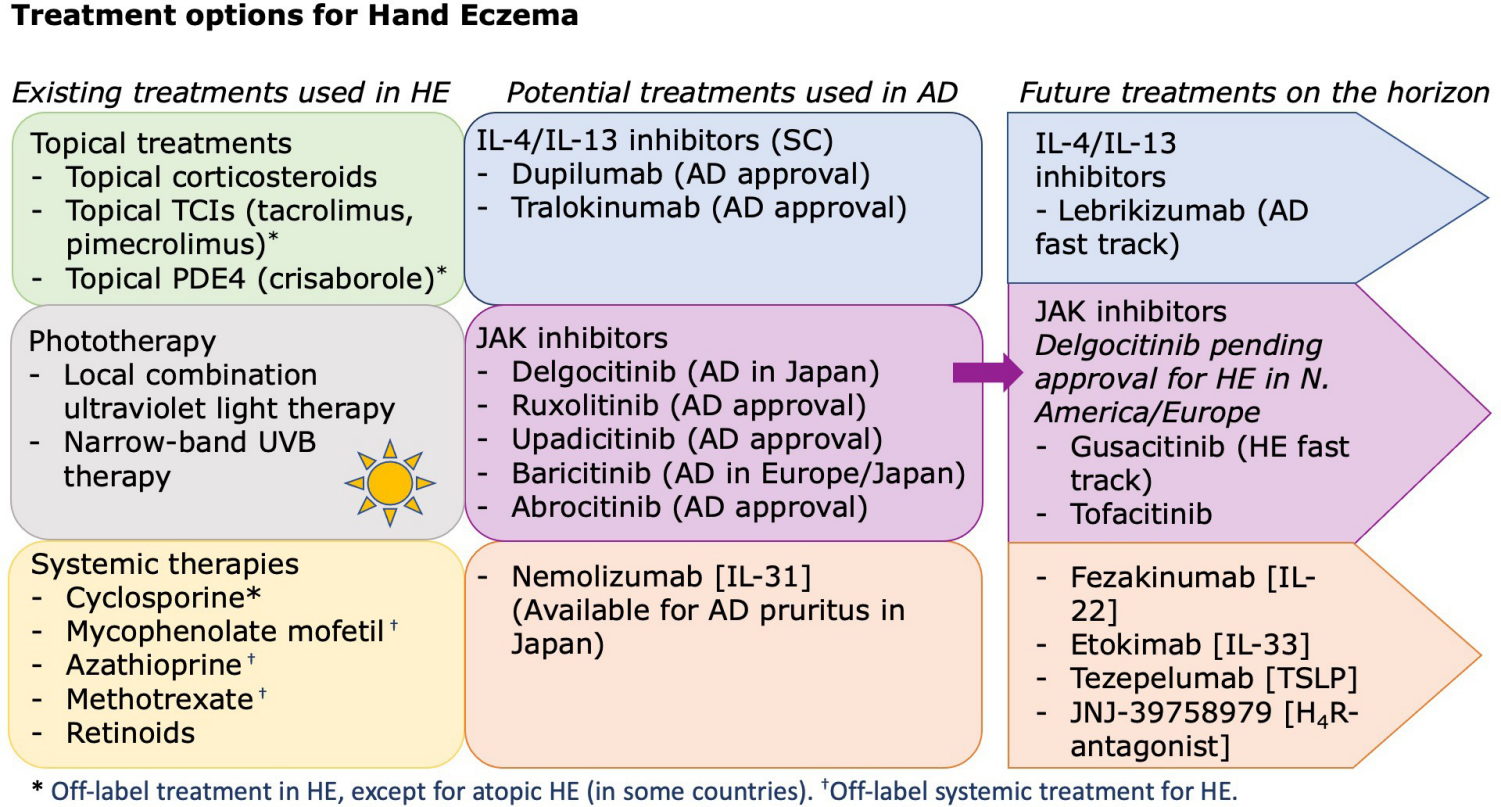
Existing and potential treatment options for hand eczema.

Hand eczema is a chronic condition that affects an estimated 14.5% of the general population.^1,2^ It has a variable presentation and may often be characterized by dry, scaly, erythematous and pruritic hand lesions that can be accompanied by vesicles, erosions, edema and hyperkeratosis. It has severe quality of life (QoL) ramifications in those that struggle with it, including days missed from work or school, productivity loss and impaired work functioning.^3^ For years, the standard of care included topical moisturizing creams, topical steroids and more recently systemic agents. As new therapeutic targets emerge and recent advances are being developed, it is now more possible than ever that hand eczema can be managed via the underlying mechanisms instead of simply symptomatically. In this review, we explore the current and emerging treatments of hand eczema and explore future directions to how this condition may be managed in clinical practice (Figure 1).

## Methods

This structured review explores the current treatments of hand eczema and new treatments on the horizon. A review of the literature was conducted to identify current treatment options for hand eczema and chronic hand eczema. The terms ‘hand eczema’, ‘hand dermatitis’ were used to search PubMed, CENTRAL and Embase. To identify new therapies still undergoing investigation, we used the terms ‘hand eczema’, ‘hand dermatitis’, ‘atopic dermatitis’, and ‘vesicular eczema of hands and/or feet’ to search Clinicaltrials.gov for all studies until December 2022.

## Pathogenesis of Hand Eczema

The aetiology of hand eczema is multi-factorial, including a complex interplay of genetics, environment, and exposures. Epidermal barrier dysfunction plays an essential role in the development of hand eczema, resulting in altered moisture retention and increased sensitivity and irritation of the skin.^4^ The role of the epidermal barrier includes protecting the skin from water loss and penetration of allergens and irritants. The three most common triggers for the development of hand eczema are an atopic predisposition, contact allergies or irritant damage.^4^ One of the primary genetic basics identified in those with an atopic predisposition include the Filaggrin (FLG) gene.^5,6^ This is the same gene that underlies other atopic conditions such as atopic dermatitis (AD), asthma, allergic rhinitis and certain food allergies.^7,8^ The increased permeability of the skin barrier allows allergic contact sensitization to occur causing allergic contact dermatitis (ACD) of the hands, as well as increased penetrance of irritants to create the basis of irritant contact dermatitis (ICD). Filaggrin mutations may increase susceptibility to hand eczema that is characterized by both ACD and ICD.^9^ However, loss of function (LoF) FLG gene do not seem to be associated with hand eczema in adults without AD though these mutations may predict early onset of hand eczema in patients with AD.^10-12^ Chronic hand eczema is defined as a dermatitis that has lasted more than 3 months or relapses at least twice per year.^13^

Classification of hand eczema is often difficult and controversially discussed. Recent approaches include etiological factors as well as clinical subtypes.^14^ The European guidelines suggested to differentiate between ICD, ACD, atopic hand eczema and protein contact dermatitis or contact urticaria^14^ and also feature clinical subtypes like hyperkeratotic or acute recurrent vesicular hand eczema, nummular hand eczema and pulpitis.^14^ ICD arises from continued exposure to irritants that compromise the skin barrier such as detergents, solvents, sweat and frequent water exposure.^15^ ACD is a type IV hypersensitivity reaction to a chemical substance such as nickel, chromate, rubber or preservatives.^15^ Atopic hand eczema is the most common form and plays an important role as a risk factor for the development of hand eczema as up to 50% of patients with chronic hand eczema have other forms of atopic diseases such as asthma, allergic rhinitis, and atopic dermatitis.^15^ Occupational hand dermatitis provides a significant challenge to clinicians as patients are often not able to avoid certain allergen or irritants. This often results in prolonged sick leave and loss or change of job.^16^ Hand eczema has been shown to have a prevalence of up to 40% in certain occupations, like health care workers or professions doing manual work.^16^ Given the complexity of these factors at play, the management of hand eczema can be challenging for clinicians as it is not a single disease mechanism that can be targeted to improve symptoms, but instead various possible processes that contribute to the clinical picture.

### Molecular Pathways of Hand Eczema

The immunologic processes that underlie the pathophysiology of hand eczema vary according to the main triggers. Each of ICD, ACD and atopic hand eczema have their own immune signature.^15^ ICD shows a Th1/Th17 immune profile that is a result of the activation of IL-1A, IL-1B, tumor necrosis factor (TNF)-α, granulocyte-macrophage colony-stimulating factor (GM-CSF) and IL-8 with exposure to the irritant.^15^ Immune profiles differ in ACD based on the allergen and various signatures have been described based on metals, fragrance and rubber allergens.^15,17^ Skin barrier dysfunction in both AD and hand eczema is associated with a reduction in ceramides in the stratum corneum as well as the expression of enzymes involved in lipid metabolism.^18^ Cells such as *T* helper 2 (Th2) and Th22 are overexpressed in the skin of patients with AD, and they have been shown to alter lipid and protein content, down-regulate tight junctions and inhibit antimicrobial peptides (AMPs) which regulate the skin barrier.^18^ Meanwhile, structural barrier proteins such as FLG, FLG-2 and hornerin (HRNR) are downregulated in hand eczema.^19^ The Th2 and Th22 cells also produce cytokines such as Interleukin (IL)-1, -5 and −13. The major effects of these cytokines include immunoglobulin E (IgE) class switching, increased Th2 cell survival and downregulating affected skin antimicrobial peptides (AMPs) such as human beta-defensin 2 (HBD-2) and single human cathelicidin peptide LL-37.^20^ Upregulation of antimicrobial peptides like S100A7, S100A8, S100A9 has been described which may be a potential indicator of increased microorganism penetration and inflammation and so has downregulation of desquamation-related enzymes which may reflect hyperkeratosis.^19^ Hyperkeratosis in patients with hand eczema occurs when contact allergens and/or irritants trigger the release of keratinocyte-derived cytokines such as TNF-α.^20^ This triggers inflammation and epidermal hyperproliferation which results in thickened skin and the formation of scale.^21^ The release of IL-17 by Th1 and Th2 lymphocytes also cause the activation of IL-4 and interferon-γ activation of keratinocyte proliferation.^22^ In those with an atopic predisposition, there is also the activation of Janus kinase (JAK) signalling pathways. Pruritus is particularly present in those with an AD component of their hand eczema and is associated with the IL-31 cytokine released by Th2 cells.^18^

### Existing Therapies

#### Topical therapies

Skin hydration and use of emollients remains the basis of every treatment for hand eczema given that the disruption of the epidermal barrier is at the forefront of disease pathogenesis. In addition, the avoidance of allergens and irritants is paramount to reduce disease exacerbation. All of these provide a foundation to ensuring treatment success of hand eczema with pharmacotherapeutic treatments.^23^ The mainstay of pharmacotherapy of hand eczema for the past few decades has been topical corticosteroids (TCSs). These can be titrated based on potency to achieve adequate remission of hand eczema flares and used more sparingly as maintenance treatments.^23^ However long-term use of TCSs can be associated with various adverse effects (AEs) including skin atrophy and alteration of the epidermal tight junction (TJ) leading to further epidermal barrier dysfunction.^24^ Because of this, they should be used only cautiously and are not suitable for long-term use.^25^

Other topical therapies include the use of topical calcineurin inhibitors (TCIs) such as tacrolimus or pimecrolimus.^26,27^ The use of these TCIs have also been shown to reduce pruritus in addition to their anti-inflammatory effect. TCIs are licensed for atopic dermatitis and not for hand eczema of other etiologies.^28^ It has been suggested that pimecrolimus may be not as effective for hand eczema given the difference in skin permeability of the palms compared to other parts of the body.^14^

Other approved topical treatments for AD such as crisaborole, which is a phosphodiesterase-4 (PDE4) inhibitor are being used in atopic hand eczema, although not available everywhere. Crisaborole regulates the production of inflammatory cytokines through the degradation of cyclic adenosine monophosphate.^29^ Its low molecular weight allows increased penetration of the skin and its topical nature decreases the potential for adverse systemic effects.^30^ Its effectiveness has previously been shown in AD in both children and adults.^31^ A retrospective chart review of 251 patients further suggests that it may be an effective treatment for hand AD with 72.2% of patients having an improvement of symptoms, 38.9% being “clear” and 61.1% being “clear” or “almost clear”.^32^

Physical therapies such as UV(ultraviolet light) phototherapy have also previously been used to treat hand eczema second-line to topical treatments in clinical practice.^33^ Local psoralen plus UVA therapy (PUVA) has been reported to improve investigator-rated control of hand eczema compared to narrow-band UVB therapy in hand eczema.^26^ While effective, given the need for frequent visits to ensure treatment success, this modality can often be a challenge for patients.^34^

#### Systemic therapies

Systemic therapies are indicated for severe hand eczema that has failed topical treatments. Systemic treatments include oral steroids, retinoids, or immunosuppressive therapies such as cyclosporin, methotrexate, mycophenolate mofetil and azathioprine.

Among systemic treatments used for hand eczema, only oral steroids and retinoids have been widely accepted. Systemic steroids are often used in flares but are not suitable for long-term management given potential adverse effects.^14^ Alitretinoin has been approved for hand eczema refractory to potent topical steroids in Canada, South Korea and several European countries.^18,35^ Alitretinoin was shown to have positive effects with “clear” or “almost clear” status achieved in half of the patients with hand eczema in a trial of 24 weeks, it is now licensed and commercially available to treat hand eczema in Canada.^36^ As with other retinoids, there are various AEs to consider including teratogenicity, photosensitivity, skin dryness, and headaches.^36^

Cyclosporine, an oral calcineurin inhibitor that inhibits T-cell-dependent immune responses is used widely as a systemic treatment for AD in Europe.^37^ While it has a fast onset of action, it can be associated with AEs such as nephrotoxicity and hypertension with long-term use.^38^ It has been shown to be effective in the treatment of hand eczema in a retrospective daily use study, with about 62.9% of patients having a good response to treatment at 3 months, particularly in those with vesicular hand eczema.^39^ In a retrospective study in South Korea, it has also been shown to have good response in 40.9% of patients though this was compared to 68.2% of patients on oral alitretinoin.^40^

Methotrexate (MTX) is an S-phase-specific drug that primarily works by interfering with DNA synthesis through inhibition of the dihydrofolate reductase enzyme. MTX has also been shown to work on the adenosine pathway exerting anti-inflammatory effects and is widely used for other dermatological diseases such as psoriasis.^41^ Cells with a high rate of turnover such as the keratinocytes of the epidermis, the hair matrix, the gastrointestinal tract and bone marrow are more susceptible to its action and are also often the first to show its toxicity.^42,43^ There are no clear studies that establish its effectiveness in the treatment of hand eczema, though there are reports of positive effects in 37% of patients in a Dutch retrospective study (N = 42) as well as among 12 patients with 40% clearance or almost clearance after 12 months.^44-46^

Mycophenolate mofetil (MMF) is a reversible inhibitor of the enzyme inosine monophosphate dehydrogenase (IMPDH) to prevent de novo purine synthesis and has been used off-label in the treatment of autoimmune conditions.^47^ It has been used for severe cases of AD with reported partial or full remission at about 77.6% of cases.^48^ AEs with the use of MMF were generally transient or mild, with headaches, gastrointestinal upset, abdominal discomfort, diarrhea and constipation.^48^ A small proportion of cases experienced hematological effects including anemia, leukopenia, neutropenia and thrombocytopenia.^48^ There are currently no unique studies for MMF in the management of hand eczema.

Azathioprine is an immunosuppressive metabolite that inhibits purine synthesis and immunoglobulins such as IgG and IgM.^18^ It also causes the accumulation of 6-thioguanine nucleotides in lymphocytes which inhibit the expression of endogenous inflammatory mediators such as TNF-related apoptosis inducing ligand, TNF receptor superfamily member 7 and α4-integrin. Adverse effects of azathioprine include lymphocytopenia and other abnormalities in cell count.^49^ It is not licensed for hand eczema, but has shown to be effective in treating hand eczema and chronic foot eczema when used in combination with a topical therapy such as clobetasol propionate 0.05%, with an improvement in the Hand Eczema Scoring Index (HECSI) of 91.29% in the group with adjunct azathioprine compared to 64.66% in the group with topical clobetasol propionate 0.05% only at 24 weeks of treatment.^50^ In addition a randomized trial of patients with severe AD, azathioprine was shown to have a mean relative reduction in the severity scoring of AD in 39% (Standard deviation [SD], 25%) compared to methotrexate which had a 42% reduction (SD, 18%) at 12 weeks.^51^

### Emerging Therapies

There were 56 ongoing clinical trials identified for pharmacological treatments for hand eczema on Clinicaltrials.gov from 2000 - 2022, with 16 that are new or ongoing (Supplemental Table 1). These included studies for dupilumab, ruxolitinib, delgocitinib (LEO124249), gusacitinib (ASN002), AFX 5931, and roflumilast (ARQ-252). Given the overlap between some of the pathophysiology of hand eczema and AD, emerging therapies for AD are starting to cross-over and might gain approval for the treatment of hand eczema in the future. Two major classes of drugs emerging for the treatment of hand eczema include IL-4/IL-13 inhibitors and JAK inhibitors.

## IL-4/IL-13 Inhibitors

IL-4/IL-13 function to increase the levels of IgE and eosinophils and have been implicated in the pathogenesis of atopy.^52^ The inhibition of IL-4/IL-13 reduces the T_H_2 response to improve pruritic immune-mediated inflammatory dermatosis.^53^

Dupilumab is a human monoclonal IgG4 antibody that inhibits IL-4 and IL-13 signal transduction by binding the shared α-subunit of the IL-4 receptor that has been shown to be effective in AD.^54^ It has been shown to have positive effects on patients with AD that also had hand eczema, with mean HECSI score reductions of 49.2 points (range 0 – 164, 95% within subject confidence interval 46.4-52.0) and the achievement of 75% on the HECSI achieved by 60%.^55,56^ It has also been suggested to potentially be helpful in ACD given the overlap in immune mechanisms shared with AD as specific allergens such as nickel have been shown to activate both innate and adaptive immunity (via the TH1 and TH17 mediated pathways), while fragrances and rubber may activate primarily a TH2 mediated pathway.^17,57^ It is currently still undergoing Phase 2 clinical trials for the treatment of hand eczema specifically (Supplemental Table 1), though case series and observational studies have shown positive off-label indications for hand eczema.^55,56,58,59^ Dupilumab has also shown to be effective in ACD as well as for non-atopic causes of hand eczema such as occupational ICD.^60-63^ The most frequent AEs reported were nasopharyngitis and headaches, and skin infections were found to be more frequent in the placebo group.^54^ Dupilumab-induced ocular surface disease is another important consideration for dermatologists as it has been shown to be more frequent in patients with AD when compared to patients with asthma, chronic rhinosinusitis with nasal polyps, or eosinophilic esophagitis.^64^ Given the limited number of studies in hand eczema specifically, there have not been specific reports on increased incidence of this adverse effect in patients with this condition and the mechanism of action of this aggravation in patients with AD is still unclear.^65^

Tralokinumab is a human monoclonal antibody that inhibits IL-13 which recently been approved for AD.^66^ The Phase 2b studies were two identically designed 52 week randomized, double-blind placebo-controlled trials showing an improvement of EASI and IGA scores at week 12 in the tralokinumab groups. The percentage of participants on tralokinumab achieving EASI 75 was 25% in one cohort, and 33.2% in the second compared to 12.7% and 11.4% respectively in placebo.^66^ Phase 3 trials of two identically designed 52 week randomized, double blind trials (ECZTRA 1 and 2), at week 16 patients on tralokinumab that had an IGA score of 0 or 1 was 15.8% compared to 7.1% in the placebo group for ECZTRA 1 and 22.2% compared to 10.9% in ECZTRA2.^66^ The majority of AEs appeared to be mild or moderate with the most common to occur in the tralokinumab group compared to the placebo group being upper respiratory tract infections (URTIs), conjunctivitis, though overall it was well tolerated.^66,67^ There have not yet been formal studies in hand eczema, but further use in AD may catalyze its use for hand eczema specifically.^68^

Lebrikizumab is a subcutaneous monoclonal IL-13 antibody that prevents the formation of IL-13R Rα1/IL-14Rα heterodimer receptor signaling complex.^69^ It has been found to have positive dose-dependent results in a Phase 2 a study as well as a favorable safety profile in a Phase 2b trial.^69,70^ Phase 3 studies found that the percentage of patients achieving an IGA score of 0 or 1 and a reduction of 2 or more points from baseline at week 16 were 43.1% (95% CI 37.1 to 49%) in the lebrikizumab group compared to 12.7% (95% CI 7.0 to 18.5%) in the placebo group and this was similar in the second Phase 3 trial with 33.2% in the lebrikizumab group compared to 10.8% in placebo.^71^ There were low rates of treatment adverse effects (TEAEs) which were most commonly URTIs, nasopharyngitis, headache, injection site pain and fatigue.^69^ Follow-up studies for AD are in progress.

## JAK-Inhibitors

JAK-inhibitors target the JAK-cytoplasmic signal transducer and activator of transcription factors (STATs) pathway to prevent the mediation of the cytokine pathways associated with inflammatory signals.^72^ Four different JAKs have been identified as potential targets including JAK1, JAK2, JAK3 and TYK2.^72^ The function of these are closely coupled with STATs which include STAT1, STAT2, STAT3, STAT4, STAT5A/STAT5B and STAT6.^73^ While it is unclear if JAK inhibitors directed towards AD will also be available for hand eczema, it has been suggested that because of their T_H_2 suppression they may potentially benefit ACD where various allergens have been shown to cause a T_H_2 skew.^17,74,75^ An overview of current JAK inhibitors is presented in Supplemental Table 2.

### Topical JAK-Inhibitors

Delgocitinib is a pan-JAK inhibitor that inhibits all members of the JAK family (JAK1, JAK2, JAK3 and tyrosine kinase 2).^38^ JAKs play a role in the activation of immune cell signaling and keratinocytes, and their inhibition suppresses the overactivation of the skin’s inflammatory response.^76^ Delgocitinib may also improve skin barrier function by suppressing STAT3 activation and promoting an increase in levels of terminal keratinocyte differentiation proteins such as filaggrin to improve skin barrier function.^77^ Delgocitinib ointment has been approved for the treatment of AD in Japan.^78^ A Phase 2 a study showed improvements in HECSI scores at week 8 for delgocitinib participants compared to vehicle.^79^ AEs were reported in 57% of delgocitinib and 45% vehicle groups, with the most common being nasopharyngitis, wound infection after biopsy.^79^ Among those in the vehicle group, 29% experienced a worsening of hand dermatitis compared to 5% in the delgocitinib ointment group.^79^ A Phase 2b dosing study of delgocitinib cream at 1 mg/g, 3 mg/g, 8 mg/g and 20 mg/g compared to vehicle group was recently completed in the United States, Denmark and Germany.^80^ Results reported include improvements in Investigator’s Global Assessment (IGA) scores for hand eczema (IGA-HE) of “clear” or “almost clear” in 21.2% at 1 mg/g, 7.8% in 3 mg/g, 36.5% in 8 mg/g, 37.7% in 20 mg/g and 8% in the vehicle group.^80^ AEs included nasopharyngitis, headache, eczema (reported most commonly in those receiving delgocitinib vehicle cream, 16%).^80^ However, the majority of patients enrolled in the study were identified as white, which may limit the generalizability to patients of other ethnicities and skin types.^80^ Currently, delgocitinib cream is undergoing clinical trials for the management of hand eczema (Supplemental Table 1). Phase 3 trials showed and IGA-CHE score of 0 or 1 with an at least 2 point improvement from baseline in 19.7% of patients receiving twice daily delgocitinib cream compared to 9.9% in vehicle (*P* = .006) in the DELTA 1 study.^81,82^

Ruxolitinib is a selective JAK1 and JAK2 inhibitor that is a topical treatment. In AD, it has been shown to significantly reduce pruritus. Improvement in EASI of 71.6% and of IGA in 38.0% at week 4 was described.^83^ Most common AEs included nasopharyngitis, URTI, headache and application site burning which occurred in both vehicle treated and ruxolitinib cream subjects.^84^ Topical ruxolitinib cream was undergoing two Phase 3 clinical trials for the treatment of HE but these have been withdrawn as of November 2022.

Tofacitinib is a topical small-molecular JAK inhibitor that decreases the JAK-STAT signal in keratinocytes and inhibits IL-4.^85,86^ Its effectiveness has been shown in plaque psoriasis and AD.^87-89^ In AD, the mean EASI score was significantly improved in patients receiving tofacitinib (−81.7%) compared to those treated with vehicle (−29.9%) at week 4.^89^ Its oral form has been approved for the treatment of rheumatoid arthritis, psoriatic arthritis, ulcerative colitis and polyarticular course juvenile idiopathic arthritis.^90^

### Oral JAK-Inhibitors

Gusacitinib (also known as ASN002) is an oral JAK/spleen tyrosine kinase (SYK) inhibitor that selectively blocks JAK1/JAK2/JAK3 as well as TYK2 and SYK, it also targets the Th1, Th2, Th17 and Th22 cytokine pathways and SYK-mediated IL-17 signaling in keratinocytes.^91^ It has been investigated in a Phase 2 trial for hand eczema with promising results including dose-dependent improvements in hand eczema compared to placebo, with a 72% improvement in HECSI compared to 20.8% in placebo (Supplemental Table 2).^92^ Those treated with 80 mg of gusacitinib also showed a 69.5% reduction in total lesion symptom score (TLSS) compared to 49% that received a lower dose (40 mg).^92^ It has previously been shown to improve moderate to severe AD, with dose-dependent changes to EASI and only accompanied by mild side effects such as headache, nausea, diarrhea and nasopharyngitis being the most common and back pain, mild hypertension and low lymphocytes being less commonly reported AEs.^91^

Upadicitinib is an oral selective JAK1 inhibitor. A Phase 2b study in AD showed that there were higher mean improvements from baseline in EASI scores in the upadicitinib group and that this followed a dose response curve from 7.5 mg, 15 mg and 30 mg compared to placebo (39% [6.2%], *P* = .03; 62% [6.1%], *P* < .001; and 74% [6.1%], *P* < .001 vs 23% [6.4%]).^93^ The most commonly reported AEs were URTI, worsening AD and acne.^93^ In a study comparing upadacitinib to dupilumab in moderate to severe AD, at week 16 it was found that 71% of patients that received upadacitinib and 61% of patients that received dupilumab achieved EASI75. AEs such as serious infection, eczema herpeticum, herpes zoster, and laboratory-related adverse events were higher for patients who received upadacitinib, though rates of conjunctivitis and injection-site reactions were higher in dupilumab patients.^94,95^ Early results suggest that upadacitinib may also be an effective treatment for improvement in hand eczema in patients with AD, with treatments of upadacitinib 15 mg (UPA15) and 30 mg (UPA30) showing reductions from baseline HECSI scores as early as week 1 and with improvements of 68% (UPA15) and 74% (UPA30) in HECSI at week 16.^96^

Baricitinib is an oral selective and reversible inhibitor of JAK1 and JAK2 which possesses some affinity for JAK3 and TYK2.^97^ It was initially approved for the treatment of rheumatoid arthritis but has since been shown to be effective in AD.^98^ Recently, case reports have suggested it is effective for hand eczema, resulting in decreased clinical symptoms though no formal clinical trial investigations have yet been conducted.^99^

Abrocitinib is an oral JAK1 inhibitor which reduces IL-4 and IL-13 signaling.^100^ In a Phase 3 trial double-blind trial of patients with AD warranting systemic therapy, in addition to topical therapy; patients were randomized to receive abrocitinib 200 mg or 100 mg orally once daily, dupilumab 300 mg subcutaneously every other week (with a loading dose of 600 mg), or placebo.^100^ IGA response at week 12 was shown in 48.4% of patients treated with 200 mg of abrocitinib, 36.6% in those treated with 100 mg abrocitinib, 36.5% in those treated with dupilumab and 14% in the placebo group.^100^ The most commonly reported AEs were diarrhea, nausea, viral URTI, and headache.^101^

Other treatments being investigated for AD which may soon be available for hand eczema include nemolizumab (anti-IL-31), fezakinumab (anti-IL-22), etokimab (anti-IL-33), and JNJ-39758979 (H_4_R-antagonist).^102^

## Discussion

Hand eczema is a chronic and challenging condition to manage. Topical first-line therapies are often insufficient for managing severe disease, and while existing systemic therapies have shown overall success, they are often accompanied by more serious AE profiles. Delgocitinib may be a promising alternative topical therapy as it has already been approved for the use of AD in Japan and is slated to come to the Canadian and European markets specifically for hand eczema.^78^

In the landscape of emerging pharmacotherapeutic options for AD, there are now new biologics and systemic treatments which are starting to cross-over for the treatment of hand eczema. Though there have not been very many specific hand eczema trials, the promise of potential success in the context of AD allows us to be hopeful they may rejoin a clinician’s treatment toolbox in the near future. We have highlighted two major classes of pharmacotherapy including biologics such as IL-4/IL-13 inhibitors as well as JAK inhibitors. Clinicians should be aware of new advances in AD as they might translate later to the treatment of hand eczema, with current ongoing trials among various JAK inhibitors specifically for the treatment of hand eczema. Of note, prior studies have identified that only around 36.5% of patients with severe hand eczema receive systemic therapy as treatment.^103^ The advent of newer topical therapies may bring in a different clinical landscape for both clinicians and patients whereby there are more topical options available before beginning a systemic therapy for hand eczema. Meanwhile both patient and physician hesitancy to start a systemic treatment will continue to further decrease with new innovations in treatments and safety to allow a more patient-centered approach to management of hand eczema.

### Future Directions

It is an exciting time for clinicians that are involved in treating patients with hand eczema as there is an acceleration of available treatments that can be offered. In addition to existing conventional topical and oral treatments, there is an increasing pool of innovative systemic treatments that target the molecular mechanisms of hand eczema pathogenesis directly. With the increase in efficacy seen with these new drugs, we are also noting improved adverse effect profiles, making them attractive options to add to a clinician’s management toolbox for patients with hand eczema. Clinicians and patients should be aware of these new developments as well as emerging therapies as the direction of hand eczema treatment is advancing at an unprecedented pace.

## Supplemental Material

Table S1 - Supplemental material for A Review of Existing and New Treatments for the Management of Hand Eczema Click here for additional data file. Supplemental material, Table S1, for A Review of Existing and New Treatments for the Management of Hand Eczema by Jessica S. S. Ho and Sonja Molin in Journal of Cutaneous Medicine and Surgery
