# Supplementary material for: A Review of Existing and New Treatments for the Management of Hand Eczema
Source: J Cutan Med Surg. 2023 Jul 27;27(5):493–503. doi: 10.1177/12034754231188325 (PMC10617006; doi:10.1177/12034754231188325)
Supplement: Table S1 - Supplemental material for A Review of Existing and New Treatments for the Management of Hand Eczema [file sj-pdf-1-cms-10.1177_12034754231188325.pdf]

Supplementary Materials

Table S1. New pharmacological treatments for hand eczema listed on Clinicaltrials.gov (as of December 2022)

NCT Number	Title	Participants (N)	Intervention	Age	Phases	Primary outcome	Blinding	Study Completion
NCT04512339	Dupilumab in Severe Chronic Hand Eczema (DUPSHE)	30	Dupilumab	18 years to 75 years	Phase 2	Hand Eczema Severity Index (HECSI) at week 16	Double (Participant, Investigator)	01-Dec-22
NCT03861455	Efficacy and Safety of Dupilumab Chronic Hands Eczema Refractory to Highly Potent Topical Corticosteroids (DUPECZEMAIN)	94	Dupilumab	18 years and older	Phase 2	Severity score mTLSS (modified Total Lesion Symptom Score) at week 16	Double (Participant, Investigator)	01-Jun-22
NCT04417894	A Study to Evaluate the Efficacy and Safety of Dupilumab in Adult and Adolescent Patients With	130	Dupilumab	12 years and older	Phase 3	Investigator's Global Assessment (IGA) hand and	Quadruple (Participant, Care Provider, Investigator,	25-May-23

	Moderate-to-Severe Atopic Hand and Foot Dermatitis (Liberty-AD-HAFT)					foot score of 0 or 1 at 16 weeks	Outcomes Assessor)	
NCT05293717	Topical Ruxolitinib in Chronic Hand Dermatitis	15	Ruxolitinib	18 years to 75 years	Phase 1/ Phase 2	Hand Eczema Severity Index (HECSI) at week 16	Double (Participant, Investigator)	31-Dec-23
NCT02664805	Proof of Concept, Twice Daily Applications of LEO 124249 Ointment in the Treatment of Chronic Hand Eczema	91	LEO 124249 (delgocitinib) ointment	18 years to 65 years	Phase 2	Physician's Global Assessment (PGA) at visit 6 (56 days)	Quadruple (Participant, Care Provider, Investigator, Outcomes Assessor)	Oct-16
NCT03683719	Phase 2b Dose-ranging Trial to Evaluate Delgocitinib Cream 1, 3, 8, and 20 mg/g Compared to Delgocitinib Cream	258	Delgocitinib cream	18 years and older	Phase 2	IGA-CHE at week 16	Double (Participant, Investigator)	20-Apr-20

	Vehicle Over a 16-week Treatment Period in Adult Subjects With Chronic Hand Eczema							
NCT04871711	Efficacy and Safety of Delgocitinib Cream in Adults With Moderate to Severe Chronic Hand Eczema (DELTA 1)	470	Delgocitinib cream	18 years and older	Phase 3	IGA-CHE at week 16	Double (Participant, Investigator)	26-Dec-22
NCT04872101	Efficacy and Safety of Delgocitinib Cream in Adults With Moderate to Severe Chronic Hand Eczema (DELTA 2)	450	Delgocitinib cream	18 years and older	Phase 3	IGA-CHE at week 16	Double (Participant, Investigator)	02-Jan-23
NCT04949841	Open-label Multi-site Extension Trial in Subjects Who Completed the	600	Delgocitinib cream	18 years and older	Phase 3	IGA-CHE and HECSI at week 16	None (Open Label)	Sep-23

	DELTA 1 or DELTA 2 Trials (DELTA3)							
NCT05355818	Efficacy and Safety of Delgocitinib Cream in Adolescents 12-17 years of Age With Moderate to Severe Chronic Hand Eczema (DELTA TEEN)	92	Delgocitinib cream	12 to 17 years	Phase 3	IGA-CHE treatment success at Week 16	Double (Participant, Investigator)	17-Oct-23
NCT05259722	A 24 Week Trial to Compare the Efficacy and Safety of Delgocitinib Cream 20 mg/g Twice-daily With Alitretinoin Capsules Once-daily in Adult Participants With Severe Chronic Hand Eczema	510	Delgocitinib cream	18 years and older	Phase 3	HECSI score at Week 12	Single (Outcomes Assessor)	25-Sep-23

NCT03703895	A Study for a Topical Medication Versus Placebo in Patients With Hand Dermatitis (AFX5931)	20	Topical AFX5931	12 years and older	Phase 4	IGA and HECSI at week 16	Triple (Participant, Care Provider, Outcomes Assessor)	04-Apr-19
NCT03728504	Study to Evaluate ASN002 in Subjects With Moderate To Severe Chronic Hand Eczema	97	ASN002	18 years to 75 years	Phase 2	Total Lesion Symptom Score (mTLSS) at week 16	Quadruple (Participant, Care Provider, Investigator, Outcomes Assessor)	28-Apr-20
NCT04378569	Safety and Efficacy of ARQ-252 Cream in Subjects With Chronic Hand Eczema	230	ARQ-252 cream 0.1%/ 0.3%	18 years and older	Phase 1/ Phase 2	IGA score at Week 12	Triple (Participant, Care Provider, Outcomes Assessor)	24-Feb-21

Table S2. JAK-inhibitor therapies studied in hand eczema

JAK-inhibitor	Target	Route	Study status	Approvals
Delgocitinib	Pan-JAK inhibitor (JAK1, JAK2, JAK3, tyrosine kinase 2)	Topical	Phase 3 for hand eczema, Phase 3 completed for AD	Approved for AD in Japan<br>Approval for hand eczema in Europe/ North America pending
Ruxolitinib	Selective JAK1 and JAK2 inhibitor	Topical	Phase 3 for hand eczema, Phase 3 completed for AD	Approved for AD
Tofacitinib	JAK-STAT signalling and IL-4 inhibitor	Topical	No trials for hand eczema, Phase 2 for AD	Not approved for AD or hand eczema
Gusacitinib	Selective JAK1/JAK2/JAK3 inhibitor and TYK2/SYK inhibitor	Oral	Phase 2 for hand eczema, Phase 2b for AD	Fast-track designation for approval in hand eczema
Upadacitinib	Selective JAK1 inhibitor	Oral	No trials for hand eczema, Phase 3 completed for AD	Approved for AD<br>Supplemental investigation of hand eczema in AD patients of Phase 3 trial[96]

Baricitinib	Selective JAK1/JAK2 inhibitor	Oral	No trials for hand eczema,<br><br>Phase 3 completed for AD	Approved for AD in Europe and Japan
Abrocitinib	JAK1 inhibitor	Oral	No trials for hand eczema,<br><br>Phase 3 completed for AD	Approved for AD
